# The Dual Role of the Gut Microbiota in Cancer Chemoresistance

**DOI:** 10.1002/mbo3.70357

**Published:** 2026-07-08

**Authors:** Hamed Tahmasebi, Aisa Bahar, Meisam Khazaei, Mohammad Reza Arabestani

**Affiliations:** ^1^ School of Medicine Shahroud University of Medical Sciences Shahroud Iran; ^2^ Department of Bacteriology Pasteur Institute of Iran Tehran Iran; ^3^ Biochemistry Department Faculty of Medicine Iran University of Medical Sciences Tehran Iran; ^4^ Department of Microbiology School of Medicine Hamadan University of Medical Sciences Hamadan Iran; ^5^ Nutrition Health Research Center Institute of Health Sciences and Technologies Hamadan University of Medical Sciences Hamadan Iran

**Keywords:** cancer, chemoresistance, dysbiosis, fecal microbiota transplantation, gut microbiota, probiotics, tumor microenvironment

## Abstract

Chemoresistance is one of the primary reasons that cancer chemotherapy fails to deliver successful treatment outcomes and contributes to poor overall survival rates for patients with cancer. New research has begun to shed light on the effects of the gut microbiome (GM). This new research will examine how certain microorganisms (referred to as “bad bacteria”) can contribute to cancer treatment failure, as well as how others (such as *Bifidobacterium*, *Akkermansia*, and *Lactobacillus*) can enhance treatment success. This review will focus on the molecular mechanisms underlying these effects, including drug metabolism by microorganisms, modulation of the immune system by microorganisms, regulation of cellular apoptosis by microorganisms, and metabolic crosstalk between tumor tissue and the microbiome. Finally, we will look at new therapies under development that leverage knowledge of the microbiome to combat chemoresistance, including fecal microbiota transplantation, targeted probiotic and prebiotic supplementation, and dietary modifications. By studying the complex interactions among the host, the microbiome, and chemotherapeutic agents, we hope to demonstrate how microbiome‐centered approaches can tailor and enhance an individual's cancer treatment while transforming the GM from a passive participant to an active target in cancer therapy.

## Graphical abstract

Comparison between Microbiota and Cancer therapy.

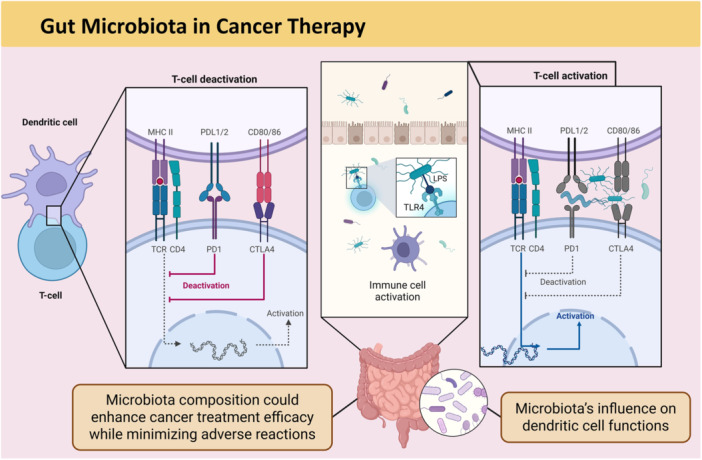

**Abbreviations**
5‐FU: 5‐fluorouracil
AOM/DSS: azoxymethane/dextran sulfate sodium
CDA: cytidine deaminase
CRC: colorectal cancer
CTLA‐4: cytotoxic T‐lymphocyte–associated protein 4
FMT: fecal microbiota transplantation
GSDME: gasdermin E
HDAC: histone deacetylase
ICI: immune checkpoint inhibitor
KD: ketogenic diet
LDH: lactate dehydrogenase
LPS: lipopolysaccharide
NLR: NOD‐like receptor
NSCLC: nonsmall cell lung cancer
PD‐1: programmed cell death protein 1
PD‐L1: programmed death‐ligand 1
PRR: pattern recognition receptor
PyNP: pyrimidine nucleoside phosphorylase
SCFA: short‐chain fatty acid
TLR: toll‐like receptor
TLS: tertiary lymphoid structure
TME: tumor microenvironment
ZO‐1: zonula occludens‐1

## 1 Introduction

Chemotherapy has been a key part of many different types of cancer treatment for decades, and while many advances have been made to improve cancer treatment, chemotherapy resistance (which can either be inherited or developed) may present the most challenging obstacle to treating cancer (Sung et al. 2021). Resistance to chemotherapy often leads to unsuccessful treatment, cancer recurrence, and decreased patient survival rates (Vasan et al. 2019; Geller et al. 2017). Chemotherapy resistance remains an area of intense research and is thought to involve a range of factors, including human genetic variation, differences in tumor biology, and alterations in the metabolism of chemotherapy drugs in cancer cells (Holohan et al. 2013). However, as research continues, there is a growing body of scientific literature that suggests that the gut microbiome (GM) (which is defined as the many trillions of microorganisms that are colonizing the human gastrointestinal tract) plays an essential role in determining how well a patient responds to cancer treatment and their overall clinical outcome (Roy and Trinchieri 2017; Zitvogel et al. 2018).

The human gut microbiome (HGM) comprises a large and functionally diverse community of microorganisms, and its overall diversity and composition are crucial to the host's health, including nutrient metabolite production, gut barrier integrity, and the development and function of the immune system (Lynch and Pedersen 2016; Neurath et al. 2025). Eubiosis is the state in which the microbiota are in balance, and dysbiosis refers to an imbalance in the composition and/or function of the gut microbiota, leading to the development of numerous diseases spanning the spectrum of inflammatory bowel diseases, metabolic disorders, and malignancies, among others (Levy et al. 2017). New research has compellingly shown that HGM can significantly impact the effectiveness and/or toxicity of various anticancer therapies, including chemotherapy and immunotherapy, by modulating the intestinal microbiota (Levy et al. 2017; Zhao et al. 2023).

The gut microbiota's influence is not straightforward; it can both enhance and undermine the effectiveness of chemotherapy. Chemoresistance in tumors can come from certain types of bacteria in the gut, which are considered “bad bacteria.” These bacteria produce substances that may directly metabolize or deactivate chemotherapy drugs, alter the tumor microenvironment (TME) to make it less supportive of immune cell attack on cancer cells, or activate pathways in tumor cells that prevent drug‐induced cell death (Brennan and Garrett 2019; N. Wang et al. 2024). By contrast, there are also “good bacteria” in the gut, which help enhance the effectiveness of chemotherapy through the production of substances that stimulate both systemic and local immune responses against cancer cells, produce potentially cytotoxic metabolites to kill tumor cells directly, and enhance the host's tolerance to chemotherapy, as they reduce the side effects associated with chemotherapy (Do et al. 2025; Ohtani et al. 2023).

The purpose of this review is to thoroughly examine the dual role of the gut microbiota in combating cancer chemotherapy resistance. The first focus will be on how cancer treatment disrupts the intricate balance of bacteria and damages the intestinal lining. We will then examine the methods used by harmful bacteria (especially *Fusobacterium nucleatum*) for developing chemoresistance. Following that, we will highlight the protective/sensitizing roles of healthy bacteria (*Bifidobacterium* and *Akkermansia muciniphila*). The review will also present data describing the interplay among microbial metabolites, host immune systems, and the tumor microenvironment. Lastly, this review will critically analyze new treatment options that manipulate the GM, including fecal microbiota transplantation (FMT), probiotics, and dietary changes, to overcome chemotherapy resistance and achieve personalized cancer therapy. The knowledge gained from exploring this whole microbial–host–drug axis will provide new possibilities for improving therapeutic outcomes for patients with cancer all over the world.

## 2 The GM and Its Disruption by Cancer Therapy

This intricate relationship is called eubiosis. It is important to note that this relationship is highly sensitive to disruption by various factors, with cancer chemotherapy among the strongest disruptors (Montassier et al. 2015). The cytotoxic effects of cancer chemotherapeutic agents, designed to target rapidly dividing cancer cells, inadvertently damage other rapidly dividing cells in the host, including those lining the intestine, thereby drastically altering the microbiota (van Vliet et al. 2009). This, in turn, triggers a series of events that can affect the treatment's outcome, including the potential induction of chemoresistance.

### 2.1 Chemotherapy‐Induced Gut Dysbiosis

One of the major effects of chemotherapy is the induction of significant gut dysbiosis. It is generally defined by reductions in microbial α‐diversity, which comprises richness and evenness in microbial community structure, and alterations in the proportions of dominant bacterial phyla (Montassier et al. 2015; S. Li et al. 2024). Empirical evidence shows that chemotherapeutic drugs reduce the proportion of beneficial commensals, especially those belonging to the Firmicutes phylum, such as butyrate‐producing *Clostridium*, and increase the proportion of pathogenic microbes, especially those belonging to the Proteobacteria phylum, such as *Escherichia coli* (van Vliet et al. 2009; S. Li et al. 2024). This shift is detrimental to the beneficial effects of the normal microbiota composition and creates a tumor‐promoting environment in the gut (Taur et al. 2014). The loss of commensals impairs the production of important metabolites, including short‐chain fatty acids (SCFAs), which are essential for colonocyte health and function, thereby further potentiating the detrimental effects of the chemotherapeutic treatment (Hajjar et al. 2024).

### 2.2 Compromise of the Intestinal Barrier

**Figure 1:**
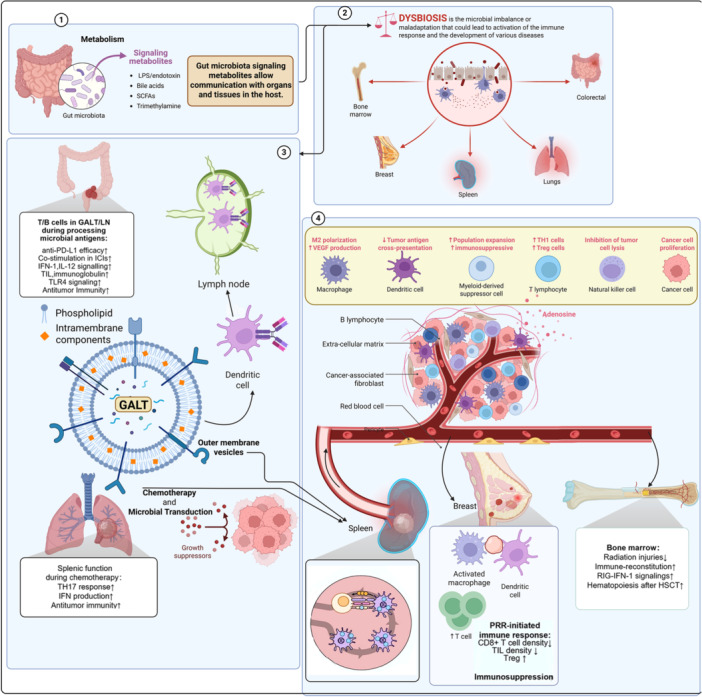
Interactions between the gut microbiota and cancer development. (1, 2) The gut microbiota can establish contact with the intestinal mucosa, resulting in genotoxic effects, stimulation of the proliferation of epithelial cells, disturbance of polarity, and metaplasia of the intestines. The microbiota could activate hematopoiesis within the thymus and bone marrow via the RIG‐I–IFN‐1 pathway after hematopoietic stem cell transplantation (HSCT) and have a radioprotective effect during radiotherapy. (3) The gut microbes and their metabolites or OMVs act upon the GALT, lymph nodes, and spleen through regulation of T cells and DCs, for example, by promoting the TH17 response, production of interferons, antigen presentation, and signaling through IFN‐1, IL‐12, and TLR4. (4) Gut microbes and tumor‐associated microbes can also interact with the TME, having an immunostimulatory effect by presenting microbe‐related antigens to T cells or inducing immunosuppression through modulation of the ratio between regulatory T cells (Tregs) and tumor‐infiltrating lymphocytes (TILs). The role of microbiomes and OMVs in affecting the TME can also be shown in the presence of microbe‐related metabolites and OMV‐mediated cargo. Microbiomes secrete various agents that can influence the activity of innate immune cells, such as TLR4, neutrophils, TNF‐α, and reactive oxygen species (ROS), thereby promoting tumorigenesis and shaping the activity of the adaptive immune system by stimulating co‐stimulation of T cells described above. CTL, cytotoxic T‐lymphocyte; DC, dendritic cell; GALT, gut‐associated lymphoid tissue; ICI, immune checkpoint inhibitor; IFN‐1, type I interferons; IL‐12, interleukin‐12; LN, lymph node; LPS, lipopolysaccharide; MDSC, myeloid‐derived suppressor cells; NK, natural killer; OMVs, outer membrane vesicles; PD, programmed death; PRR, pattern recognition receptor; RIG‐I, retinoic acid‐inducible gene I; SCFAs, short‐chain fatty acids; TH, T helper cell; TLR4, toll‐like receptor 4; TME, tumor microenvironment; TNF‐α, tumor necrosis factor‐α; VEGF, vascular endothelial growth factor.

The intestinal barrier is a complex, multilayered system that separates the lumen of the gastrointestinal tract from the host's internal environment. The physical barrier of the intestinal barrier consists of a mucus layer and intestinal epithelial cells, which are tightly connected by tight junctions, while the immunological barrier comprises immune cells in the lamina propria (Neurath et al. 2025). Chemotherapy affects all layers of this barrier, weakening it by thinning the mucus layer, directly damaging intestinal epithelial cells, and altering tight junction complexes (van Vliet et al. 2009; Dahlgren and Lennernäs 2023). The proteins ZO‐1 and Occludin are critical for maintaining these tight junction complexes, which are required to close the paracellular space. Downregulation and dysfunction of these proteins, induced by chemotherapy, increase intestinal permeability, a phenomenon referred to as “leaky gut” (Ulluwishewa et al. 2011; Marchiando et al. 2010). This phenomenon enables bacteria and their components, such as lipopolysaccharide (LPS), from the gut lumen to enter the systemic circulation, leading to a systemic inflammatory response syndrome, which may result in severe drug‐induced toxicities, as well as a pro‐inflammatory environment that can induce chemoresistance and metastasis (van Vliet et al. 2009; Almonte et al. 2026) (Figure 1).

### 2.3 The Gut–Liver Axis and Systemic Consequences

Impairment of gut barrier function also has significant systemic effects, particularly through the gut–liver axis. The liver is the primary organ to be exposed to the products transported from the gut into the systemic circulation. The entry of various microbial compounds, such as LPS, can trigger an immune response in the liver, particularly in Kupffer cells, leading to liver inflammation (Seki and Schnabl 2012). This type of chronic inflammation can significantly affect liver metabolic function, especially the ability to process chemotherapy agents, which is important for chemotherapy outcomes (Q. Li and Liu 2025). In addition, the products of gut microbial metabolism can affect the liver. For example, alterations in bile acid (BA) metabolism, which is largely regulated by the gut microbiota, are known to affect the progression of liver cancer and the outcome of immunotherapy (Ma et al. 2018). Therefore, chemotherapy‐induced dysbiosis and impairment of gut barrier function create a cycle in which local gut effects drive systemic inflammation, which can negatively affect the outcome of chemotherapy across the organism (Abdulaal et al. 2025).

## 3 The “Dark Side”: Microbiota‐Mediated Chemoresistance

While a dysbiotic gut generally creates a pathogenic environment, certain microbes can directly impair the effectiveness of chemotherapy through various complex mechanisms. Such microbes are termed “oncomicrobes” or “pathogenic bacteria” and include bacteria capable of inactivating drugs through metabolism, manipulating host cell signaling to evade apoptosis, and creating immunosuppressive environments, thereby contributing significantly to chemotherapy failure (Brennan and Garrett 2019).

### 3.1 Direct Microbial Metabolism of Chemotherapeutic Drugs

One of the most direct ways in which bacteria can cause chemoresistance is by enzymatic degradation of chemotherapy drugs. A classic example is the inactivation of gemcitabine, a cornerstone of therapy for pancreatic and various other solid cancers (Mendes and Vale 2024). Gemcitabine is a nucleoside analog that, once incorporated into DNA, interferes with DNA replication and causes programmed cell death. However, some bacteria, particularly those in the Gammaproteobacteria class (e.g., *E. coli*) (Geller et al. 2017; G. Xu et al. 2025), can produce a long form of cytidine deaminase (CDA). This bacterial form of CDA effectively inactivates gemcitabine by converting it into 2′,2′‐difluorodeoxyuridine (dFdU) before it can act on cancer cells. There is evidence that these bacteria are commonly present in pancreatic cancers and are associated with significant resistance to gemcitabine. Importantly, however, resistance induced by these bacteria can be overcome by cotreatment with antibiotics, such as ciprofloxacin, which can kill these bacteria and restore drug effectiveness (Geller et al. 2017; Mendes and Vale 2024). Along these same lines, mycoplasmas have been shown to produce a pyrimidine nucleoside phosphorylase enzyme, which can indirectly contribute to gemcitabine inactivation by deamination (Mendes and Vale 2024). This “enzymatic shield” is a significant cause of acquired resistance to chemotherapy drugs.

### 3.2 Indirect Mechanisms: The Case of *F. nucleatum*

*F. nucleatum* is an important organism in the development of resistant cancers (colorectal cancer [CRC]) (Brennan and Garrett 2019; Kostic et al. 2012). It is a Gram‐negative, anaerobic bacterium typically found in the mouth. However, the presence of *F. nucleatum* has been associated with a poorer prognosis in CRC. *F. nucleatum* has developed several strategies to protect cancer cells from the effects of chemotherapy (Risoen et al. 2025).

#### 3.2.1 Modulation of Host Cell Death Pathways

Chemotherapeutic drugs primarily work by inducing apoptosis in cancer cells. *F. nucleatum* has been shown to interfere with this process. Previous studies have shown its ability to induce chemoresistance in CRC patients by activating autophagy, a cellular process of recycling. It is believed to work as a survival mechanism for cancer cells, thereby inhibiting apoptosis (N. Wang et al. 2024; Yu et al. 2017).

Recently, a new study has shed light on a previously unknown pathway that suppresses pyroptosis (N. Wang et al. 2024). Pyroptosis is a type of inflammatory programmed cell death mediated by the gasdermin family. Chemotherapeutic drugs, such as oxaliplatin and 5‐fluorouracil (5‐FU), can induce apoptosis by activating Caspase‐3. Gasdermin E, expressed in cells, is cleaved by activated Caspase‐3, releasing its N‐terminal domain (Y. Wang et al. 2017). It creates membrane pores that induce cellular swelling and lysis, thereby triggering pyroptosis. A landmark study has shown that the bacterium *F. nucleatum* inhibits pyroptosis induced by chemotherapeutic drugs. It activates the effector protein of the Hippo signaling pathway, yes‐associated protein, which upregulates the antiapoptotic protein B‐cell lymphoma 2 (BCL2). High levels of BCL2 inhibit Caspase‐3 activation, which is required to induce pyroptosis (N. Wang et al. 2024; Y. Wang et al. 2017). As a result, the entire pyroptotic cascade is blocked. By blocking the entire drug‐induced apoptosis cascade, the bacterium provides a survival advantage to the CRC cells. It directly contributes to chemoresistance. The presence of the bacterium has been positively correlated with the recurrence of CRC after chemotherapy (N. Wang et al. 2024).

#### 3.2.2 Creation of an Immunosuppressive Tumor Microenvironment

In addition to its direct effects on cancer cells, *F. nucleatum* is also known to render the TME less responsive to treatment. It can interact with host immune cells via its surface adhesins, leading to infiltration of tumor‐associated myeloid cells and the establishment of an immunosuppressive environment that protects the tumor from immune surveillance (Brennan and Garrett 2019; X. Li et al. 2025). This effect not only supports tumor growth but can also affect the success of both chemotherapy and immunotherapy, which depend on the immune system to eliminate dying cancer cells (Zhu et al. 2024). By regulating this complex interplay between cancer cell survival and immune suppression, *F. nucleatum* is a classic example of the role one “bad” bacterium can play in the development of cancer chemoresistance (Table 1).

**Table 1:** Microbiota mediated chemoresistance mechanisms.

Mechanism category	Specific mechanism/bacterial species	Description of chemoresistance	Drugs affected	Key molecular/cellular pathways	Tumor types	References
**Direct microbial metabolism**	Bacterial enzymes (e.g., β‐glucuronidases, cytidine deaminase)	Bacteria express enzymes that chemically modify and inactivate chemotherapeutic drugs before they can reach their target, or reactivate toxic metabolites, leading to resistance and increased toxicity.	**Irinotecan**, **5‐fluorouracil (5‐FU)**, Gemcitabine	Hydrolysis of drug‐glucuronides, deamination	Colorectal cancer (CRC), Pancreatic cancer	(Chang et al. (2020); Sadeghloo and Sadeghi (2025); Sun et al. (2025))
	*Citrobacter freundii* (PreTA enzyme)	*C. freundii* metabolizes 5‐FU via the PreTA enzyme (homologous to human dihydropyrimidine dehydrogenase), inactivating the drug and driving chemoresistance.	**5‐Fluorouracil (5‐FU)**	PreTA‐mediated drug inactivation	Pancreatic cancer	(Zhang et al. (2024); Tintelnot et al. (2025))
**Indirect mechanisms: The** ***Fusobacterium nucleatum*** **case**	*Fusobacterium nucleatum* (autophagy activation)	*F. nucleatum* activates the innate immune TLR4/MYD88 pathway in cancer cells, which in turn suppresses apoptosis and activates autophagy, promoting survival during chemotherapy.	Various agents	TLR4, MYD88, autophagy pathway	Colorectal cancer, Breast cancer	(Li et al.(2024); Lan et al. (2025))
	*Fusobacterium nucleatum* (ferroptosis inhibition)	*F. nucleatum* upregulates GPX4 expression via the E‐cadherin/β‐catenin/TCF4 pathway, which inhibits ferroptosis (an iron‐dependent form of cell death), thereby inducing resistance to certain chemotherapies.	**Oxaliplatin**	E‐cadherin/β‐catenin/TCF4, GPX4	Colorectal cancer (CRC)	(Li et al. (2024))
**Modulation of host cell death pathways**	Inhibition of ferroptosis	Specific microbiota can block non‐apoptotic cell death pathways, such as ferroptosis, which is crucial for the efficacy of certain drugs, thereby allowing cancer cells to survive treatment.	**Oxaliplatin**	GPX4, iron metabolism, lipid peroxidation	Colorectal cancer (CRC)	(Li et al. (2024))
	Activation of Protective Pathways (e.g., Autophagy)	By triggering cytoprotective autophagy in tumor cells, bacteria enable tumor cells to withstand chemotherapy‐induced stress, leading to treatment failure.	Various agents	Autophagy, TLR4/MYD88	Breast cancer, Colorectal cancer	(Li et al. (2024); Lan et al. (2025))
**Creation of an immunosuppressive TME**	Metabolite‐mediated immunosuppression (e.g., Lactic Acid, SCFAs)	Bacterial metabolites, such as lactic acid and short‐chain fatty acids (SCFAs), accumulate in the TME, directly suppressing the activity of cytotoxic T cells and NK cells while promoting the expansion of immunosuppressive cells, such as regulatory T cells (Tregs).	Various agents, including Oxaliplatin and Immunotherapies	T‐cell receptor signaling, JNK/c‐Jun/p38 phosphorylation, Treg induction	Triple‐negative breast cancer (TNBC), Melanoma, and other solid tumors	(Lan et al. (2025))
	Induction of Immune Checkpoint Molecules	The acidic, metabolite‐rich environment fostered by the microbiota can upregulate immune checkpoint molecules, such as PD‐L1, on immune and tumor cells, further dampening the anti‐tumor immune response and contributing to resistance.	Various agents, including Immunotherapies	MCT1/NF‐kB/COX‐2 pathway, PD‐L1	Various	(L. Wang et al. (2025))

Abbreviations: COX‐2, cyclooxygenase‐2; GPX4, glutathione peroxidase 4; JNK, c‐Jun N‐terminal kinase; MCT1, monocarboxylate transporter 1; MYD88, myeloid differentiation primary response 88; NF‐kB, nuclear factor kappa B; NK, natural killer; PD‐L1, programmed death‐ligand 1; TCF4, transcription factor 4; TLR4, toll‐like receptor 4; TME, tumor microenvironment.

## 4 The “Bright Side”: Microbiota‐Mediated Chemosensitization

In stark contrast to the harmful effects of oncomicrobes, several studies have underscored the importance of beneficial commensal bacteria to enhance the therapeutic potential of anticancer treatments. These beneficial bacteria, termed “good bacteria” or “probiotics,” can potentiate cancer therapy through various mechanisms, including promoting antitumor immunity, synthesizing cancer‐suppressing metabolites, and maintaining gut integrity to minimize systemic toxicity (Do et al. 2025; Gopalakrishnan et al. 2018) (Table 2).

**Table 2:** A brief exposition on the effects of antineoplastic drugs on the gut microbiome in the context of different cancer types.

Cancer type	Chemotherapy regimen	Effects on gut microbiota	References
Lung cancer	Pemetrexed	A reduction was observed in the Ruminococcaceae bacterial family, which produces short‐chain fatty acids (SCFAs). A substantial augmentation in the prevalence of Enterobacteriaceae and Enterococcaceae, both opportunistic bacterial families, was recorded.	(Pensec et al. (2020); Kenneth et al. (2025))
Stage III CRC	Capecitabine‐Oxaliplatin (CapeOx)	In cases of chemotherapy‐associated diarrhea, opportunistic Klebsiella pneumoniae was the predominant microorganism, occurring in 31% of cases.	(Deng et al. (2018); Kenneth et al. (2025))
CRC	5‐FU + oxaliplatin	It was consistently noted that patients receiving this course of therapy displayed a significant and marked increase in the presence and growth of Veillonella dispar, Bacteroides plebeius, and Prevotella copri within their biological systems.	(Deng et al. (2018); Kenneth et al. (2025))
Breast, colorectal, esophageal, laryngeal, and melanoma	Capecitabine, cisplatin/5‐FU, FOLFOX4, FOLFOX6, FOLFIRI, 5‐FU/folinic acid, paclitaxel, carboplatin, and gemcitabine	The GM of individuals undergoing cytotoxic therapy demonstrated a scarcity of beneficial bacteria (e.g., Lactobacillus spp., Bacteroides spp., Bifidobacterium spp., and Enterococcus spp.) and an elevated representation of opportunistic species, such as Staphylococcus spp. and Escherichia coli.	(Stringer et al. (2013); Kenneth et al. (2025))
Ovarian cancer	Paclitaxel, carboplatin, and cisplatin	The GM of individuals with ovarian cancer changes, specifically with increases in Bacteroidetes and Firmicutes and a decrease in Proteobacteria following antineoplastic treatment.	(Otto‐Dobos et al. (2024); Kenneth et al. (2025))
Breast cancer	Taxane, cyclophosphamide, carboplatin, and doxorubicin	Cognitive impairment observed during chemotherapy is associated with altered gut microbial composition, manifesting as taxonomic shifts in Fusicatenibacter, Faecalibacterium, Erysipelotrichaceae UCG‐003, Bacteroides, and Subdoligranulum.	(Otto‐Dobos et al. (2024); Kenneth et al. (2025))
CRC	5‐FU	The numbers of Deltaproteobacteria, Firmicutes, and Coriobacteria decreased, accompanied by an upregulation of mRNA expression of inflammatory cytokines such as TNF‐α, IL‐6, IL‐1β, and IL‐10, and of nitric oxide synthase in the gastrointestinal tract.	(Yixia et al. (2021); Kenneth et al. (2025))
Pancreatic ductal adenocarcinoma (PDAC)	Gemcitabine	The pharmaceutical compound reduced Gram‐positive Firmicutes from approximately 39% to 17% and decreased Gram‐negative Bacteroidetes from 38% to 17%, demonstrating comparable reductions in both groups.	(Kong et al (2019); Kenneth et al. (2025))

Abbreviations: CRC, colorectal cancer; FOLFIRI, folinic acid, fluorouracil and irinotecan; FOLFOX, folinic acid, 5‐fluorouracil and oxaliplatin; GM, gut microbiome; IL, interleukin; mRNA, messenger RNA; TNF‐α, tumor necrosis factor‐α.

### 4.1 “*Bifidobacterium*”: A Potent Immune Adjuvant

**Figure 2:**
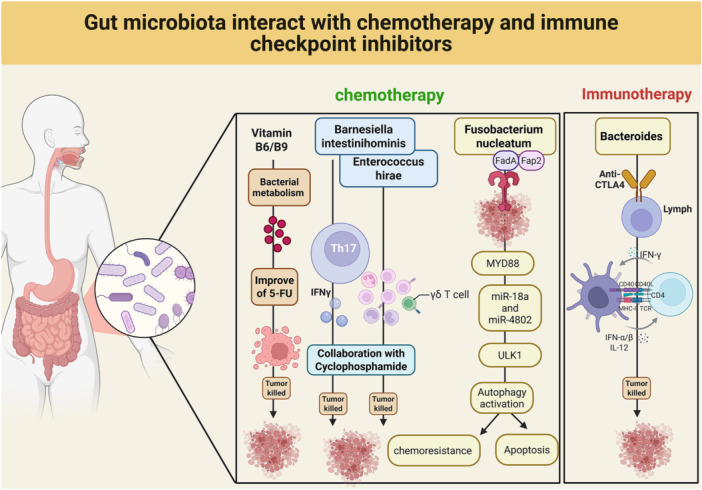
The exploration of how GMs interact with anticancer medications has become a vital research focus, uncovering how distinct bacterial communities can alter the effectiveness of chemotherapy and immune checkpoint inhibitors (ICIs). Alterations in the metabolism of ribonucleotides and vitamins B6 and B9, driven by particular bacterial mechanisms, play a crucial role in determining the effectiveness of 5‐fluorouracil (5‐FU). Evidence suggests that inhibiting bacterial deoxynucleotide metabolism can substantially enhance the therapeutic effects of 5‐FU. Certain bacteria, such as *Barnesiella intestinihominis* and *Enterococcus hirae*, play a crucial role in enhancing the antitumor efficacy of cyclophosphamide; their absence may lead to drug resistance. The migration of *E. hirae* occurs from the small intestine to secondary lymphoid organs, where it stimulates the development of pathogenic T helper 17 (pTh17) cells, while B. Intestine gathers in the colon, facilitating the generation of γδT cells that produce interferon‐gamma (IFN‐γ) during cyclophosphamide therapy. Moreover, *Fusobacterium nucleatum* contributes to chemoresistance by interacting with TLR4 and MYD88 pathways, leading to autophagy activation through selective loss of miR‐18a and miR‐4802. In addition, ICI efficacy is influenced by *Bacteroides* species; for instance, *Bacteroides fragilis* plays a pivotal role in anti‐CTLA‐4 therapy by inducing T helper 1 immune responses and promoting dendritic cell maturation within tumors via colonization of the mucosal layer. These findings underscore the complex interplay between GM and cancer therapies, highlighting potential pathways for enhancing treatment outcomes through microbial modulation. CTLA‐4, cytotoxic T‐lymphocyte–associated protein 4; GM, gut microbiome; IL, interleukin; MYD88, myeloid differentiation primary response 88; TLR4, toll‐like receptor 4; ULK1, Unc‐51‐like autophagy activating kinase 1.

The “*Bifidobacterium*” genus is recognized as a probiotic with a primary role in the healthy development of breastfeeding infants and can provide a variety of health benefits to those who consume it. The same species can also play an important role as a partner in cancer treatment (Do et al. 2025). A multitude of studies demonstrate that some species (e.g., “*Bifidobacterium* longum” and “*Bifidobacterium* breve”) within the genus “*Bifidobacterium*” can significantly enhance the effects of chemotherapy, radiation, and, most importantly, the efficacy of immune checkpoint inhibitors (ICIs) (Do et al. 2025; Sivan et al. 2015) (Figure 2).

The principal mode of action for *Bifidobacterium* is believed to be its significant immunoregulation. These microorganisms can modulate the immune system by downregulating pro‐inflammatory cytokines, such as IL‐6 and TNF‐α, while increasing the production of anti‐inflammatory mediators. Furthermore, *Bifidobacterium* can enhance the function of important antitumor immune cells, including CD8+ T‐lymphocytes, natural killer cells (NK cells), and macrophages (Kong et al. 2025). For example, oral administration of *Bifidobacterium* resulted in enhanced tumor control in mice treated with anti‐PD‐L1 monoclonal antibodies and was associated with increased CD8+ T‐cell activation and infiltration into the tumor (Sivan et al. 2015). Moreover, *Bifidobacterium* can directly affect the fate of cancer cells by modulating signaling molecules that regulate apoptosis. For example, evidence shows that *Bifidobacterium* induces apoptosis in colon cancer cells by upregulating pro‐apoptotic signaling proteins (e.g., Bax) and downregulating antiapoptotic signaling proteins (e.g., Bcl‐2 and EGFR) (Do et al. 2025; Procaccianti et al. 2023). On the basis of these findings, *Bifidobacterium* has both primed the immune system for a vigorous antitumor response and directly stimulated cancer cell death, making it extremely powerful as a chemosensitizer and immunosensitizer.

### 4.2 *A. muciniphila*: Guardian of the Gut Barrier and Immunotherapy Enhancer

*A. muciniphila*, a distinct bacterium that breaks down mucin, inhabits the intestinal mucus layer. Reports indicate that the number of *A. muciniphila* found in the intestinal mucus layer is inversely associated with obesity and inflammation; therefore, its presence at high concentrations indicates a healthy intestine (Cani and de Vos 2017). In the field of oncology, research on *A. muciniphila* and its potential to improve the efficacy of the host's responses to cancer therapies, primarily ICIs, has increased substantially (Ohtani et al. 2023; Fan et al. 2023). Evidence from clinical trials shows that cancer patients who exhibit elevated baseline *A. muciniphila* levels within the gut are more likely to respond to anti‐PD‐1 therapies when treated for nonsmall cell lung carcinoma (NSCLC) or renal cell carcinoma (Ohtani et al. 2023).

There are a variety of ways that *A. muciniphila* benefits us. First, it promotes the integrity and function of the intestinal barrier (Cani and de Vos 2017). By supporting mucus production from goblet cells and strengthening tight junctions between epithelial cells, *A. muciniphila* helps prevent the translocation of inflammatory products from gut bacteria into the systemic circulation, thereby reducing the levels of inflammatory cytokines that can impair antitumor immunity (Cani and de Vos 2017).

Additionally, *A. muciniphila* also regulates the gut‐associated immune system. Through its direct interaction with enterocytes and immune cells in the gut mucosa, *A. muciniphila* promotes the recruitment of Th1 CCR9 + CXCR3 + CD4 + T cells that migrate to the TME to enhance the function of cytotoxic CD8+ T cells (Ohtani et al. 2023). In the context of cancer chemotherapy, the administration of *A. muciniphila* enhances the efficacy of chemotherapy by inducing the TRAIL (TNF‐related apoptosis‐inducing ligand) pathway, leading to Caspase‐3 activation and apoptosis in cancer cells (Faghfuri and Gholizadeh 2024). Overall, the ability of *A. muciniphila* to enhance systemic immunity and maintain gut homeostasis makes it an excellent candidate for the next generation of probiotics to use as an adjuvant to cancer therapy (Khalili et al. 2026).

### 4.3 *Lactobacillus* Species: Mitigating Toxicity and Promoting Gut Health

**Figure 3:**
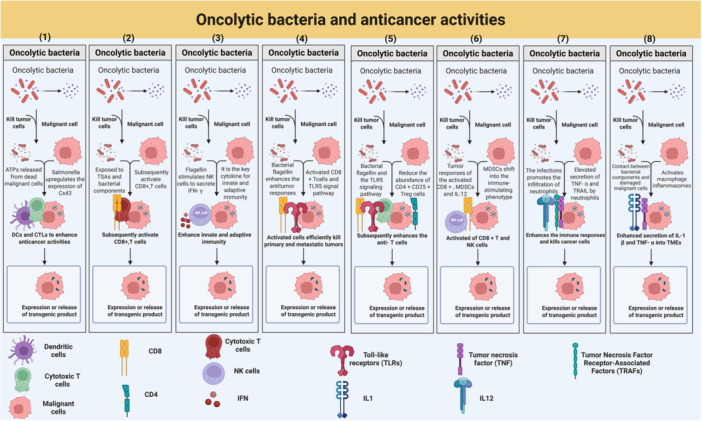
Oncolytic bacteria are emerging as a promising therapeutic strategy in cancer treatment due to their ability to mediate antitumor effects through complex interactions with the immune system and malignant cells. Upon systemic administration, these bacteria target and localize within tumor microenvironments (TMEs), initiating a cascade of immune responses that facilitate tumor regression. (1) Notably, bacteria such as *Salmonella typhimurium*, *Listeria*, and *Clostridium* directly induce apoptosis and autophagy in tumor cells, leading to the release of ATPs that recruit dendritic cells (DCs) and cytotoxic T‐lymphocytes (CTLs) to bolster anticancer activities. (2) Additionally, *Salmonella* toxins upregulate Cx43 expression, promoting gap junction formation between cancer cells and DCs for effective cross‐presentation of tumor‐specific antigens (TSAs). This antigen presentation triggers DCs to secrete pro‐inflammatory cytokines, such as IL‐1β and TNF‐α, activating CD8+ T cells to target tumor cells for destruction via perforin‐ and granzyme‐mediated cytotoxicity. (3, 4, and 5) Furthermore, bacterial flagellin enhances CD8+ T cell responses via the TLR5 signaling pathway while reducing immunosuppressive Treg cell populations. *Listeria* infections also convert myeloid‐derived suppressor cells into an immunostimulatory phenotype that boosts CD8+ T and natural killer (NK) cell activity by increasing IL‐12 production. (6) Moreover, infections by *S. typhimurium* and *Clostridium* augment neutrophil infiltration in TMEs, where elevated TNF‐α and TRAIL secretion induce apoptosis in cancer cells. (7 and 8) The interaction between bacterial components and damaged malignant cells activates macrophage inflammasomes, further enhancing secretion of IL‐1β and TNF‐α within TMEs, ultimately amplifying the antitumor immune response. Through these multifaceted mechanisms, oncolytic bacteria offer a novel avenue for harnessing the body's innate defenses against cancerous growths. ATPs, adenosine triphosphates; IL, interleukin; TLR5, toll‐like receptor 5.

Lactobacilli are various genera of bacteria that fall under the category of lactic acid bacilli. *Lactobacillus* bacteria are among the most commonly used probiotics for treating various ailments (Aboubaker 2025). *Lactobacillus* has shown promise in treating cancer by reducing the adverse effects of chemotherapy and radiation (Park 2026). Cancer patients commonly experience a plethora of negative side effects from chemotherapy. One of the most serious side effects of chemotherapy is mucositis (severe inflammation and ulceration of the mucosal surfaces of the GI tract). Many studies have shown that consuming probiotics containing *Lactobacillus* strains (including *Lactobacillus rhamnosus* GG) significantly decreases the incidence and severity of chemotherapy‐induced diarrhea and chemotherapy‐related mucositis (López‐Gómez et al. 2023; Feng et al. 2022). Probiotics are also thought to provide a healthier gut environment during the use of toxic chemotherapy by competing for space with pathogenic organisms, creating antimicrobial compounds, and improving intestinal barrier function (Sivamaruthi et al. 2020). While predominantly probiotic *Lactobacillus* strains are thought to mitigate only the negative effects of chemotherapy, some strains (such as *Lactiplantibacillus plantarum*) have shown direct anticancer activity. *L. plantarum* has been shown to induce apoptosis in cancerous cells and to produce anticarcinogenic metabolites with potential anticancer activity (Fida et al. 2025). Thus, *Lactobacillus* species can provide patients with many benefits, including increased tolerance of treatment and the establishment of a healthier gut environment (Figure 3).

## 5 Mechanisms of Microbiota–Host Interaction in the Tumor Microenvironment

The GM influences chemosensitivity beyond just the gastrointestinal tract. Gut bacteria and their products can modulate systemic immune responses using a complex, bidirectional communication network and can also directly affect the tumor microenvironment. Cancer therapy success or failure will be dictated by the extent to which these interactions occur (Lam and Goldszmid 2026; Farhadi Rad et al. 2024). Immune signaling, along with a wide range of metabolites, mediates bidirectional communication between gut bacteria and the immune system.

### 5.1 Immune Modulation: From the Gut to the Tumor

The host's immune system relies heavily on the gut microbiota for guidance and development. The host detects bacteria via microbe‐associated molecular patterns, which are recognized by pattern recognition receptors on both intestinal epithelial cells and innate immune cells (Lam and Goldszmid 2026; Le Noci et al. 2021). Thus, the continuous recognition of microbial factors in the environment continually calibrates the basic functioning of the host immune system. A healthy, diverse gut microbiota maintains immune homeostasis, whereas an imbalance (dysbiosis) disrupts TLR signaling, drives chronic inflammation, and causes immune dysregulation, all of which contribute to cancer development and treatment resistance (Giambra et al. 2023; Qing et al. 2022).

**Figure 4:**
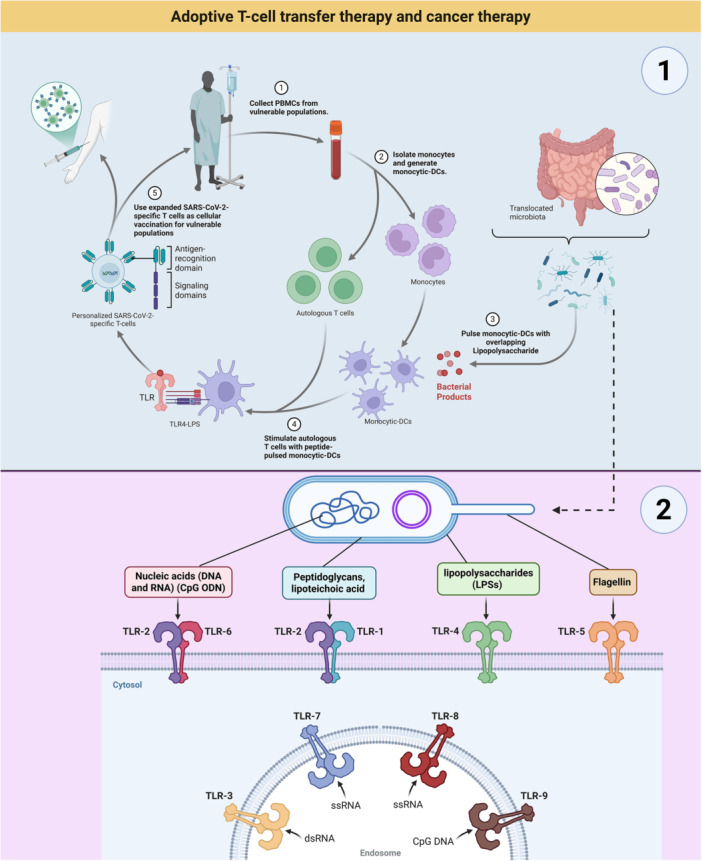
(1) Role of GM in adoptive T‐cell transfer therapy and (2) CpG oligonucleotides (ODNs) and anti‐interleukin 10 antibodies in cancer therapy. (1) The operational principles of adoptive cell therapy (ACT). ACT with tumor‐infiltrating lymphocytes (TILs) involves isolating TIL cells from peritumoral tissues, expanding them in vitro, and reintroducing them into the patient. In ACT using TCRs or CARs, T cells are harvested from a patient's peripheral blood and then genetically engineered to express TCRs or CARs, which confer the ability to selectively recognize and attack neoplastic cells once reinfused into the patient. (2) A significant advantage of bacterial treatment involves its effective induction of antineoplastic immune responses via multiple pathways. Bacterial therapeutics possess the ability to activate the innate immune system, either to combat malignant growth or to improve oncological interventions, because of the presence of pathogen‐associated molecular patterns (PAMPs)—primarily lipopolysaccharides (LPSs), peptidoglycans, lipoteichoic acid, flagellin, and nucleic acids (DNA and RNA)—which bind to pattern recognition receptors (PRRs), like Toll‐like receptors (TLRs), expressed by dendritic cells (DCs), macrophages, monocytes, and B lymphocytes. The activation of TLR4‐mediated signaling pathways9 by LPS, a predominant component of the outer membrane in Gram‐negative bacteria, drives the maturation of DCs and the M1 polarization of macrophages. On antigen‐presenting cells, TLR5 specifically detects flagellin, triggering immune and inflammatory pathways that influence tumor progression. As selective TLR2 activators, peptidoglycans, lipopeptides, and lipoteichoic acids derived from Gram‐positive bacteria alter the characteristics and activities of dendritic cells, macrophages, natural killer cells, and T cells, thereby triggering anticancer immune responses. CARs, chimeric antigen receptors; CoV, coronavirus; GM, gut microbiome; ODN, oligonucleotide; SARS, severe acute respiratory syndrome; ssRNA, single‐stranded RNA; TCRs, T‐cell receptors.

Gut‐induced immunity has a wide‐ranging effect throughout the body. Gut‐associated lymphoid tissue can prime immune cells that will become activated at different locations in the body, including tumors (Patra et al. 2025). Specialized commensal microorganisms can stimulate the differentiation and expansion of effector T cells, particularly CD8+ killer T cells and Th1 cells, both of which are required for generating anticancer immunity (Najar et al. 2026; Jing et al. 2025). The TME can also be physically altered by signals from the microbiota to promote the recruitment, expansion, and maturation of CD8+ effector T cells, which are critical for clearing tumor cells after chemotherapy or immunotherapy (Najar et al. 2026). Additionally, the gut microbiota can regulate the development of abnormal tertiary lymphoid structures (TLSs) within tumors. TLSs are ectopic collections of lymphoid tissue that act as localized centers to provide an anticancer immune response, and their presence is frequently correlated with improved cancer prognosis and response to therapy (Liu et al. 2024; Kamalabadi Farahani et al. 2025). The gut microbiota therefore regulates both systemic and local immunologic factors related to antitumor immune surveillance and coordination of antitumor immune responses through remote (i.e., from the gut to the tumor) mechanisms (Figure 4).

### 5.2 Metabolic Crosstalk: The Power of Microbial Metabolites

Small‐molecule metabolites produced by the GM can be absorbed into the bloodstream and function as signaling molecules, significantly altering host physiology and cancer biology. The most widely studied example is the production of SCFAs, including butyrate, propionate, and acetate, which result from the fermentation of dietary fiber by gut bacteria (ParsaSefat et al. 2025; Khazaei et al. 2025).

#### 5.2.1 Short‐Chain Fatty Acids

The bacteria in your large intestine ferment fibers from your diet to create SCFAs, such as acetate, propionate, and butyrate. These SCFAs are considered the primary energy for the cells lining your colon, and they also have powerful signaling abilities. Butyrate has been widely studied for its potential to prevent cancer (Hoffman et al. 2014; Bahar, Khazaei, et al. 2026). It is known to inhibit histone deacetylases (HDACs). When butyrate inhibits HDACs in cancer cells, it alters the function of some genes (a process called epigenetic modification). For example, the p21 gene, which normally inhibits the cell division cycle by causing cell cycle arrest, is reactivated by HDAC inhibition with butyrate (Khazaei et al. 2025; S. Zhang et al. 2021). This modification makes cancer cells more sensitive to chemotherapy treatment. SCFAs also play an important role in regulating the immune system. Butyrate stimulates regulatory T cells (Tregs) and enhances cytotoxic T‐lymphocytes, making them more effective and better at memory (Geng et al. 2021). A high‐fiber diet with an SCFA‐producing component has been shown to improve the response to immunotherapy among patients with melanoma, translating into better responses to chemotherapy and combined chemotherapy and immunotherapy for many patients (Khazaei et al. 2026; Hajjar et al. 2021). Thus, SCFAs mediate the microbiome's effects on cancer treatment by directly acting on cancer cells and regulating the immune response.

#### 5.2.2 Bile Acids

BAs are produced in the liver through cholesterol metabolism and serve as essential lipids involved in fat metabolism in the body. After synthesis, primary BAs are metabolized by gut microbiota into an array of secondary BAs, namely, deoxycholic acid (DCA) and lithocholic acid (LCA). Secondary BAs are powerful signal molecules that interact with several host proteins, including farnesoid X receptor and Takeda G‐protein‐coupled receptor (TGR5) (B. Han et al. 2023; L. Sun et al. 2019). The involvement of secondary BAs in carcinogenesis is not straightforward. For instance, elevated DCA levels promote carcinogenesis by inducing DNA damage, increasing reactive oxygen species (ROS) production, and activating survival mechanisms in colonic cells. Activating TGR5 can lead to tumor proliferation, especially in hepatocellular carcinoma (HCC) (Tong and Lou 2025; Gou et al. 2024). On the contrary, findings suggest that specific BAs can suppress tumors by inducing cell death (mitochondrial stress and apoptosis), particularly LCA (Gou et al. 2024; Darmadi et al. 2026). Finally, BA signaling can affect immune cell activity by altering Treg differentiation, among others (Mafe and Busselberg 2025a). Therefore, the net effect of BAs on chemoresistance depends on their composition and the cells' microenvironment, making this an interesting field of study in oncology.

#### 5.2.3 Polyamines and Other Metabolites

Polyamines, such as putrescine, spermidine, and spermine, are low‐molecular‐weight cationic compounds that play an important role in cell proliferation, differentiation, and DNA stabilization. Both the host and microbiome produce polyamines. While polyamines are crucial for cellular function, their metabolic regulation is disrupted in cancer cells, leading to elevated levels and enhanced tumor progression (Mafe and Busselberg 2025b). Some microbes may even provide polyamines to cancer cells, thus enabling their proliferation and causing resistance to chemotherapy. Nevertheless, polyamines appear to be indispensable for mounting antitumor immunity, as they help maintain T‐cell activity via autophagy, among other mechanisms (Mafe and Busselberg 2025b; Ohtani et al. 2023). Other metabolites produced by microbes include indoles and vitamins generated from tryptophan metabolism, which can affect host immunity and cellular functions (Bahar, Parsa Sefat, et al. 2025) (Table 3).

**Table 3:** Mechanisms of microbiota‐host interaction in the tumor microenvironment.

Category	Specific mechanism/metabolite	Detailed description	Key microbial examples	Effects in TME	References
**Overall mechanisms**	Microbiota‐host interaction (barrier, translocation, inflammation)	Gut microbes alter TME via direct colonization, OMV secretion, PRR activation (TLR4/NF‐κB), and systemic signals; dysbiosis promotes "cold" tumors while eubiosis creates "hot" tumors.	Pathobionts (*F. nucleatum*, ETBF, *P. anaerobius*); Commensals (*A. muciniphila*, *Bifidobacterium*)	↑Tumor growth/metastasis (via inflammation/angiogenesis); modulates therapy resistance/sensitivity.	(Feng et al. (2024); M. Xie et al. (2025))
**Immune modulation**	Gut‐to‐tumor immune reprogramming (innate/adaptive cells)	Pathogens suppress CD8+/NK/ILC3, expand MDSCs/Tregs/M2 TAMs (via CXCL1, toxins, IL‐10); beneficial microbes enhance DC maturation, CD8+ infiltration/activation, M1 polarization, and reduce exhaustion (via metabolites/signaling). Links strongly to ICI efficacy.	Pathogens: *F. nucleatum* (FadA/Fap2), *H. pylori*; Beneficial: *Lactobacillus*, *Bifidobacterium*, *A. muciniphila*	Immunosuppressive (cold TME) vs. antitumor (hot TME with ↑IFN‐γ/granzyme); improves or impairs immunotherapy.	(David et al. 2014; Greathouse et al. 2022)
**Metabolic crosstalk**	Short‐Chain Fatty Acids (SCFAs: acetate, propionate, butyrate)	Produced via dietary fiber fermentation; act via HDAC inhibition (↑histone acetylation, gene expression), GPCRs (GPR41/43/109A), metabolic fueling (acetyl‐CoA/TCA), or protein acylation. Context‐dependent (dose, cancer type, hypoxia).	*Faecalibacterium prausnitzii*, *Roseburia intestinalis*, *Bifidobacterium*, *Clostridium* spp.	Anti‐tumor: ↑CD8+/NK activation, apoptosis/ferroptosis in tumor cells, M1 polarization, ↓angiogenesis; Pro‐tumor (contextual): energy source in GBM/pancreatic, MDSC support. Enhance ICI/radiotherapy.	(M. Xie et al. (2025); Xiang et al. (2026))
**Metabolic crosstalk**	Bile Acids (BAs; primary → secondary, e.g., DCA, LCA)	Microbiota (7α‐dehydroxylation) converts primary BAs to secondary BAs; binds receptors (FXR, TGR5, VDR, S1PR2); modulates inflammation, ROS/DNA damage, and immune signaling (NFAT2 inhibition).	*Clostridium scindens* (DCA producer), other Firmicutes/Clostridia	Generally pro‐tumor in CRC/HCC: suppresses CD8+ effectors (↓IFN‐γ/granzyme), promotes immunosuppression/DNA damage; anti‐tumor in liver (NKT recruitment via CXCL16 in some contexts). Shape immunosuppressive TIME.	(Xie et al. (2025))
**Metabolic crosstalk**	Polyamines (putrescine, spermidine, spermine)	Dysregulated biosynthesis/uptake (ODC/AMD1); microbiota & diet elevate luminal/systemic levels; support proliferation, hypoxia adaptation (HIF1α), ROS generation; deplete arginine (ARG1 in MDSCs).	Gut biofilms/dysbiosis (*E. coli*, various fermenters); dietary sources amplified by microbes	Pro‐tumor: ↑MDSC/M2 TAM survival, Treg induction, effector T/NK suppression → cold TME & ICI resistance; depletion (DFMO + transport inhibitors) remodels to hot TME (↑CD8+/IFN‐γ).	(Holbert et al. (2022))
**Metabolic crosstalk**	Other Metabolites (e.g., inosine, tryptophan derivatives [IPA/I3A], TMAO)	Inosine (A2A receptor/Th1 shift); indole derivatives (AHR signaling, epigenetic); TMAO (PERK/GSDME pyroptosis). Act via metabolic reprogramming or receptor activation.	*B. pseudolongum* (inosine); *L. johnsonii*/*C. sporogenes* (IPA); *L. reuteri* (I3A); *Enterococcus* (TMAO)	Mixed: enhance Th1/CD8+ stemness & ICI (inosine/IPA); suppress via kynurenine/AHR (Tregs); TMAO boosts CD8+ or exhausts in context.	(Feng et al. (2024); M. Xie et al. (2025))

Abbreviations: AHR, aryl hydrocarbon receptor; AMD1, adenosylmethionine decarboxylase 1; CRC, colorectal cancer; CXC, chemokine receptor; CXCL16, CXC chemokine ligand 16; DC, dendritic cell; DFMO, difluoromethylornithine; ETBF, Enterotoxigenic Bacteroides fragilis; GBM, glioblastoma multiforme; GPCR, G‐protein‐coupled receptor; HCC, Hepatocellular Carcinoma; HDAC, histone deacetylase; HIF1α, hypoxia‐inducible factor 1‐alpha; I3A, indole‐3‐aldehyde; ICI, immune checkpoint inhibitor; IFN‐1, type I interferons; IL‐10, interleukin‐10; ILC3, group 3 innate lymphoid cell; IPA, indole‐3‐propionic acid; MDSCs, myeloid‐derived suppressor cells; NFAT, nuclear factor of activated T cells; NF‐kB, nuclear factor kappa B; NK, natural killer; NKT, natural killer T; ODC, ornithine decarboxylase; OMVs, outer membrane vesicles; PERK, protein kinase R (PKR)‐like endoplasmic reticulum kinase; ROS, reactive oxygen species; S1PR2, sphingosine‐1‐phosphate receptor 2; TAMs, tumor‐associated macrophages; TCA, tricarboxylic acid cycle; TGR5, Takeda G protein‐coupled receptor 5; TIME, tumor immune microenvironment; TLR4, toll‐like receptor 4; TMAO, trimethylamine N‐oxide; TME, tumor microenvironment; TME, tumor microenvironment; VDR, vitamin D receptor.

## 6 Therapeutic Modulation of the Gut Microbiota to Overcome Chemoresistance

In light of growing evidence on the role of the microbiota in cancer treatment, efforts have been made to control this complex ecosystem better to achieve better outcomes. This control can range from complete replacement of the community to alteration of diet and may help transform the microbiome into a powerful therapeutic agent against chemoresistance (Kamalabadi Farahani et al. 2025; Bahar, Porbaran, et al. 2025).

### 6.1 Fecal Microbiota Transplantation

The FMT procedure involves transferring stool samples from a healthy donor into an ill individual, thereby helping establish a normal gut microbial population. While initially used to treat recurrent *Clostridioides difficile* infections, FMT has gained traction as a possible means of restoring the abnormal microbial environment in cancer patients, thereby making them more responsive to therapies (Khoruts and Sadowsky 2016). According to the basic principles of this procedure, FMT helps restore healthy microbial activity, restore the intestinal barrier, and influence the host's immune response (Wekking et al. 2025).

**Figure 5:**
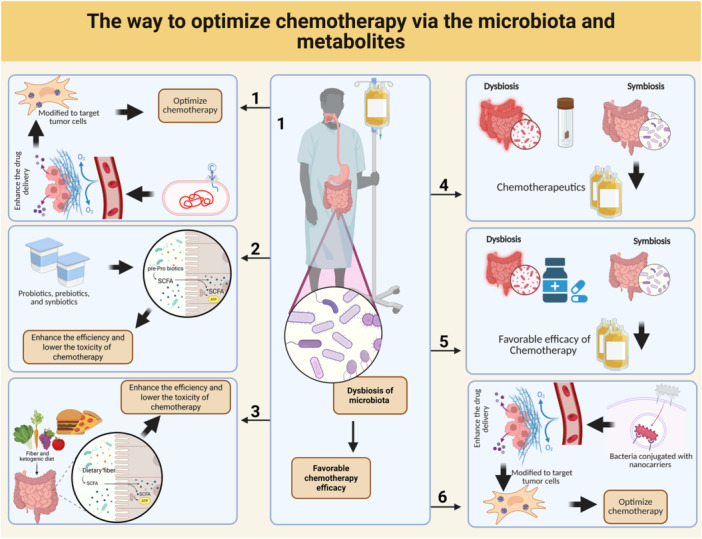
An approach to enhancing chemotherapy efficacy by utilizing the microbiota and its metabolic products. The principal approaches for leveraging the gut microbiome (GM) in cancer chemotherapy include dietary modifications, targeted antibiotic administration, administration of probiotics, prebiotics, synbiotics, fecal microbiota transplantation (FMT), genetically engineered bacteria, and bacteriophages. SCFA, short‐chain fatty acid.

Several early‐phase clinical studies have already provided convincing proof‐of‐concept evidence for this technique, especially in the context of immunotherapy. Patients with metastatic melanoma resistant to anti‐PD‐1 treatment demonstrated significant improvement in their condition following FMT from patients who responded; they experienced objective therapeutic effects (Baruch et al. 2021). Mechanistically, donor FMT increases gut microbiota diversity, upregulates beneficial bacteria, and induces desirable changes in the tumor microenvironment, including improved infiltration by CD8+ T cells (Wekking et al. 2025; H. Xu et al. 2022). Clinical studies are currently underway to determine whether FMT can reduce chemotherapy side effects and enhance its effectiveness across different types of gastrointestinal cancers (Miller et al. 2026). Despite the encouraging results achieved thus far, there are still challenges to be addressed regarding donor screening, protocol standardization, and the safety of long‐term use of this method. Nonetheless, FMT is a highly efficient way to demonstrate causality between the microbiome and therapy response and to provide a comprehensive approach to restoring the microbiome (Wekking et al. 2025) (Figure 5).

### 6.2 Probiotics and Prebiotics

Another, more direct approach would be the administration of beneficial bacteria themselves, termed probiotics, or the substrates that promote the growth of these microorganisms, referred to as prebiotics. As mentioned earlier, probiotics, including *Bifidobacterium*, *Akkermansia*, and *Lactobacillus*, show promising effects in boosting antitumor immunity and alleviating treatment‐related side effects (Do et al. 2025; Fan et al. 2023; Park 2026). Clinical studies are underway to investigate the efficacy of probiotic therapy in improving the quality of life for cancer survivors by reducing toxicity associated with chemotherapy (Feng et al. 2022; Lei et al. 2025). One phase II clinical study showed that the addition of probiotic therapy decreased chemoradiation‐induced oral mucositis in patients with nasopharyngeal carcinoma (Xia et al. 2021).

Prebiotics, mostly composed of nondigestible fibers, serve as substrates for beneficial gut microbiota, promoting the production of SCFAs, such as butyrate (Holscher 2017; Bahar, Moazzen, et al. 2026). A high‐fiber diet was found to correlate positively with progression‐free survival in patients with melanoma receiving immunotherapy, due to increased microbiome diversity and increased SCFA producers (Lei et al. 2025; Spencer et al. 2021). In synbiotics, probiotics and prebiotics are combined to provide not only the beneficial microbe but also the appropriate nutritional substrate for better colonization. Despite the benefits of “bugs‐as‐drugs,” several considerations arise when using this approach in oncology. First, efficiency is usually strain‐dependent; second, giving bacteria to immunosuppressed patients may result in bacteremia. Thus, extensive, rigorous clinical studies are needed to properly identify probiotic and prebiotic strains and dosages (J. Sun et al. 2025; Kaźmierczak‐Siedlecka et al. 2023).

### 6.3 Dietary Interventions

Dietary factors constitute some of the most powerful yet simple means of modulating the GM (Conlon and Bird 2014). The nutrients ingested determine the composition and metabolic activity of the gut microbiota, which in turn impacts cancer predisposition and treatment efficacy (David et al. 2014). Therefore, the use of diets as adjuncts to cancer therapy represents an area of much scientific and clinical interest.

One dietary factor shown to impact patient survival among those diagnosed with cancer is a prudent dietary pattern, characterized by a high intake of fiber from plant products (fruits, vegetables, and whole grains) (Greathouse et al. 2022). High‐fiber intake leads to greater microbial diversity and SCFA production, conferring anticancer and immunoregulatory functions (Spencer et al. 2021; Turner et al. 2026). Clinically, research is underway to test the ability of a high‐fiber/fermented foods‐based diet to improve gut microbial signatures and the effectiveness of cancer treatment in patients with melanoma and other cancers (Khazaei et al. 2026; Bulmer and Avenell 2025; Aslam et al. 2026).

A second dietary strategy being investigated for its role in altering gut microbiota is the ketogenic diet (KD), a diet rich in fats and very low in carbohydrates. The KD alters metabolism, leading to ketosis, which could result in glucose deprivation for tumor cells (Colombo et al. 2025). Moreover, the KD has been found to induce drastic changes in gut microbiota (Rew et al. 2022). In preclinical CRC models, KD has been shown to inhibit tumor proliferation via a mechanism involving the GM (Dimitrakopoulou et al. 2017). Specifically, KD was shown to promote colonization by bacteria producing stearic acid, a saturated fat that stimulates apoptosis in cancer cells and reduces Th17 inflammatory cell populations (Dimitrakopoulou et al. 2017). However, the application of a restrictive diet, like the KD, in clinical practice is complicated due to the poor nutritional status of many cancer patients. Still, the results provide strong evidence for the feasibility of using precision nutrition to steer the GM into an anticancer/chemosensitizing state (Greathouse et al. 2022; Colombo et al. 2025).

## 7 Evidence from Preclinical and Animal Models

Animal models, particularly those involving germ‐free (GF) (gnotobiotic) or antibiotic‐treated mice, have been instrumental in establishing a causal relationship between the gut microbiota and response to chemotherapy. These models allow researchers to dissect the specific contributions of the microbiome in a controlled environment, providing foundational evidence for translation to human studies.

### 7.1 Foundational Gnotobiotic and Antibiotic‐Depletion Studies

One of the earliest direct proofs of a link between the microbiome and chemotherapy came from pioneering studies performed by Iida et al. (2013) and Viaud et al. (2013). The use of animal models of cancer, such as sarcoma, lymphoma, and colon cancer, revealed that the anticancer activity of oxaliplatin in mice decreased significantly under antibiotic treatment or in GF conditions. This study showed that the microbiota plays a critical role in recruiting myeloid cells that produce ROS into tumors, which are essential for the therapeutic efficacy of the drugs. Recolonizing GF mice with conventional microbiota restored oxaliplatin's ability to kill cancer cells. At the same time, Viaud et al. (2013) found that the antimicrobial activity of cyclophosphamide was suppressed in antibiotic‐treated and GF mice. It was shown that the drug disrupted the integrity of the intestinal barrier, leading to the translocation of some Gram‐positive bacterial species (*Enterococcus hirae* and *Barnesiella intestinihominis*) into lymph nodes, where they stimulated the development of pathological T helper 1 cells and memory T helper 17 cells.

These groundbreaking results established that the efficacy of a particular chemotherapy depends not only on its direct cytotoxic activity but also on interactions with the host's gut microbiota to mediate antitumor immunity. Other studies have further confirmed this notion by establishing that oxaliplatin response is influenced by its ability to trigger peripheral neuropathy, which limits the dose used to treat cancers (Shen et al. 2017).

### 7.2 Studies on Specific Bacterial Taxa and Drug Interactions

Further research based on these general insights into the involvement of microbiota has led to the identification of specific bacteria and the processes they initiate. An interesting case is that of gemcitabine resistance. Animal studies have shown that tumors colonized by customer due diligence (CDD)–expressing *E. coli* can resist gemcitabine treatment. Removal of bacteria using a specific antibiotic (such as ciprofloxacin) can restore sensitivity to chemotherapy drugs, providing an efficient way to target bacterial resistance (Geller et al. 2017).

A large number of animal studies have also investigated the effect of probiotics on improving the efficacy of chemotherapy drugs while minimizing the harmful effects. The administration of *L. rhamnosus* GG (LGG) along with oxaliplatin was found to reduce tumor growth and decrease the intestinal injury in mouse models of CRC (Shen et al. 2017; Z. Zhang et al. 2025). Likewise, *Bifidobacterium* infantis was found to prevent mucositis in rat models of CRC by modulating T‐cell immunity (Mi et al. 2017). There is strong evidence for targeting these probiotic strains for specific applications.

In contrast, animal models have proved essential for identifying the ability of specific pathobionts to support tumor growth and drug resistance. CRC xenografts, when injected with *F. nucleatum*, not only showed increased tumor growth but were also resistant to treatment with 5‐FU and oxaliplatin (Kostic et al. 2013). Further research into the molecular mechanisms of resistance in this model suggests that the bacterium can activate autophagy and signaling processes in cancer cells.

### 7.3 FMT in Animal Models

In animal studies, FMT has been used to demonstrate that the chemotherapy‐responder phenotype is transmissible to other organisms. One such experiment in immunotherapy involved transferring fecal material from melanoma patients who demonstrated objective responses to anti‐PD‐1 treatment into GF mice. It was observed that recipient mice showed better tumor control than those receiving microbiota from nonresponder patients, thereby confirming a possible causal link between the two (Matson et al. 2018). Since then, the same strategy has been successfully applied to chemotherapy. Indeed, transplanting a microbiome from mice that respond positively to a specific chemotherapy drug into mice that do not respond often leads to the development of the desired phenotype in the latter group and to the restoration of chemosensitivity. Thus, such studies provide the best experimental evidence for the causal role of the complete microbial flora and have helped lay the groundwork for FMT clinical trials.

In addition, animal models may be useful in assessing the potential of combination therapies. To wit, multiple studies have evaluated the impact of FMT combined with either chemotherapy or chemo‐immunotherapy. As mentioned above, their results often point to the synergy of the two treatments. Such animal models play an important role in determining the optimal timing for FMT before chemotherapy and in identifying beneficial microbial signatures in stool donations.

## 8 Findings From Human Clinical Trials

The compelling evidence from preclinical models has spurred a wave of clinical research aimed at understanding and manipulating the gut microbiota in cancer patients. This research encompasses observational studies correlating baseline microbiota with treatment outcomes, as well as interventional trials testing the efficacy of microbiome‐modulating strategies.

### 8.1 Observational Studies: Linking Microbiome Signatures to Chemoresistance

Several clinical studies have found a link between the patient's microbial composition before commencing treatment and the effectiveness of chemotherapy. Greater baseline gut microbial diversity has been reported to enhance treatment efficacy in metastatic CRCs. Certain taxa have also been associated with differential responses to chemotherapy treatments. For instance, *Bacteroides* and Prevotella were found to elicit distinct responses to irinotecan‐based chemotherapy in metastatic CRC patients (D'Amico et al. 2021). Similarly, in an examination of a cohort of epithelial ovarian cancer patients, variations in gut microbiota composition during chemotherapy were found to influence therapeutic responses, with *Coriobacteriaceae* and *Bifidobacterium* associated with platinum resistance in some cases (D'Amico et al. 2021; Blanco et al. 2025).

In a population of triple‐negative breast cancer patients receiving neoadjuvant chemotherapy, research has shown that gut microbes may be used to predict pathological complete response (Sarfraz et al. 2024). While such correlative studies play a crucial role in generating novel hypotheses and discovering microbial biomarkers, it must be acknowledged that they do not establish causation and should be followed up by mechanistic investigations in larger cohorts.

### 8.2 Interventional Trials: Modulating the Microbiome to Improve Outcomes

The ultimate goal of this field is to translate observational findings into effective therapies. Several types of microbiome‐targeted interventions are currently being tested in clinical trials to overcome chemoresistance and reduce toxicity.

#### 8.2.1 Fecal Microbiota Transplantation

A series of phase I and II studies demonstrates that FMT from healthy, responsive donors can resensitize patients with metastatic melanoma or NSCLC who are resistant to anti‐PD‐1 therapy to subsequent anti‐PD‐1 treatment (Davar et al. 2021; Fernandes et al. 2026). For instance, FMT converted approximately 30% of nonresponders to responders, with positive immunologic changes in TME (Davar et al. 2021). While earlier attention has been paid to the application of FMT for immuno‐oncology purposes, substantial scientific evidence has emerged regarding its use in chemotherapy, encouraging studies combining FMT with chemotherapy or chemo‐immunotherapy. Among such trials, a pilot study is exploring the safety and feasibility of concomitant FMT administration during neoadjuvant chemo‐immunotherapy for esophageal cancer patients (NCT07292142). Additionally, a study is being designed to identify the effectiveness of FMT for reversing drug resistance in HCC (NCT06643533). The above‐described studies are highly significant for elucidating the safety of FMT in immunocompromised patients with cancer and for demonstrating FMT's efficacy in chemotherapy. Early studies have demonstrated that FMT is generally considered to be safe; the most frequent AEs are GI‐related and minor (Duttagupta et al. 2026).

#### 8.2.2 Probiotics, Prebiotics, and Synbiotics

Many studies have examined the use of probiotics, mainly to alleviate the adverse effects of chemotherapy on patients' digestive systems. According to a meta‐analysis of 17 studies, probiotic intervention was associated with a clear benefit, leading to a decrease in adverse reactions to chemotherapy or radiotherapy, including diarrhea and mucositis (Rodriguez‐Arrastia et al. 2021). The use of probiotics is known to help repair intestinal damage by inducing crypt and villus regeneration (Khorashadizadeh et al. 2024) (Figure S1).

While more information about the ability of probiotics to reduce the adverse effects of chemotherapy is becoming available, there are still some controversies regarding their influence on enhancing the antitumor effect of the treatment (Redman et al. 2014). Certain studies have revealed a positive impact; for example, synbiotics are believed to be helpful when given as additional therapy in CRC patients, with good surgical outcomes and reduced risk of chemotherapy side effects (Amitay et al. 2020; Mazinani et al. 2026). It should be noted that, due to the great variety in the types of probiotics used, doses, and administration periods, it is difficult to provide meaningful comparisons across studies [134]. At the same time, there exists the risk of negative interactions with chemotherapy drugs or an increased risk of infections among severely immunocompromised patients, which did not emerge in the majority of studies. There is also the ongoing NCT07044310 trial investigating the effects of probiotics on preventing bone loss in breast cancer patients who are on adjuvant treatment, as well as the NCT06088940 study on the effects of probiotics on digestive problems among cancer survivors after treatment.

#### 8.2.3 Dietary Interventions

An important clinical study of melanoma patients receiving immunotherapy found that high dietary fiber intake was associated with significantly better progression‐free survival (Spencer et al. 2021). This was due to the increased abundance of SCFA‐producing bacteria, including species of *Ruminococcaceae*. Even though this study focuses on immunotherapy rather than chemotherapy, it should be noted that the mechanisms underlying its results are relevant to both therapies, since they involve T‐cell activation and enhanced immune system function. Nowadays, several clinical trials are underway to establish standards for diet therapy in combination with chemotherapy. For example, there is interest in studying how changes in diet quality during cancer treatment affect the microbiome (C. Han et al. 2026). Though the scientific background for dietary and prebiotic‐based strategies is strong enough, more studies are needed to provide guidelines to cancer patients considering their special needs (Gardiner et al. 2025). Individual nutrition based on microbial analysis might become the next stage of development (Table 4).

**Table 4:** Evidence from preclinical and animal models of the role of the gut microbiota in cancer chemoresistance.

Category/subcategory	Study/model	Cancer type/drug	Key findings (dual role aspect)	Specific microbiota/intervention	References
**Preclinical & animal models: Foundational gnotobiotic & antibiotic‐depletion studies**	Viaud et al. (2013) – Germ‐free (GF) or broad‐spectrum antibiotic (e.g., vancomycin)‐treated mice with subcutaneous tumors	Various (e.g., MC38 colon, P815 mastocytoma)/Cyclophosphamide (CTX)	Antibiotic depletion or GF status abolishes CTX efficacy by preventing Gram+ bacterial translocation and TH17/immune priming; partial restoration via specific bacteria transfer (sensitivity‐enhancing role of intact microbiota).	Depletion of commensals (e.g., loss of Enterococcus hirae‐like species); microbiota translocation is critical for efficacy.	(Viaud et al. (2013))
**Preclinical & animal models: Foundational gnotobiotic & antibiotic‐depletion studies**	Iida et al. (2013) – Antibiotic cocktail or GF mice with subcutaneous tumors	Various solid tumors/Platinum agents (oxaliplatin, cisplatin)	Microbiota disruption impairs tumor response by reducing myeloid‐cell ROS production and altering the tumor microenvironment; intact commensals enhance platinum efficacy (sensitivity role).	Broad commensal depletion (e.g., Gram+ species).	(Iida et al. (2013))
**Preclinical & animal models: Foundational gnotobiotic & antibiotic‐depletion studies**	Multiple models (e.g., reviewed in 2025 systematic) – Antibiotic‐treated vs. conventional mice	CRC, PDAC, lung/Gemcitabine, 5‐FU, oxaliplatin	Depletion often worsens response (resistance‐promoting via lost immune/metabolic support) or, in select PDAC models, enhances gemcitabine by altering tumor proteomics; dual context‐dependent effects.	Broad‐spectrum depletion (e.g., vancomycin/ampicillin).	(Mai et al. (2025))
**Preclinical & animal models: Studies on specific bacterial taxa & drug interactions**	Li et al. (2024); Gao et al. (2025); Yu et al. (2017) – In vitro CRC cells + xenograft mouse models	CRC/Oxaliplatin (and 5‐FU)	F. nucleatum infection induces resistance via TLR4/MYD88 → autophagy upregulation (inhibits apoptosis); E‐cadherin/β‐catenin/TCF4 → GPX4 overexpression (inhibits ferroptosis); PVT1 upregulation via NF‐κB (resistance‐promoting). Human CRC tissue correlation.	Enrichment of *Fusobacterium nucleatum* (oral‐origin pathogen).	(Yu et al. (2017); B. Li et al. (2024); Gao et al. (2025))
**Preclinical & animal models: Studies on specific bacterial taxa & drug interactions**	Geller et al. (2017) & follow‐ups – In vitro + mouse models	Various (e.g., pancreatic)/Gemcitabine (and CPT‐11, CTX)	Specific taxa inactivate drugs: cytidine deaminase isoforms (reversible by ciprofloxacin); *E. coli* via dihydropyrimidine dehydrogenase (preTA operon) inactivates 5‐FU (resistance‐promoting).	*Proteus* spp., *Gamma‐Proteobacteria*, *E. coli*.	(Geller et al. (2017))
**Preclinical & animal models: Studies on specific bacterial taxa & drug interactions**	Various (e.g., *Lactobacillus plantarum* studies) – CRC cell lines + mouse models	CRC/5‐FU; lung/Cisplatin; melanoma/sarcoma/CTX	Beneficial taxa enhance cytotoxicity (e.g., reduce cancer stem cells, activate BAX/CDKN1B, boosts IL‐6/IFN‐γ, TH17 → CD8+ activation); SCFAs (butyrate) via PINK1/Parkin (sensitivity‐enhancing).	*Lactobacillus* spp. (e.g., *plantarum*, *acidophilus*), *Bifidobacterium*, butyrate‐producers (*Roseburia*).	(Zhou et al. (2017); Rezai et al. (2025); Fathi et al. (2026))
**Preclinical & animal models: Fecal microbiota transplantation (FMT) in animal models**	X. Wang et al. (2025) – LLC tumor‐bearing mice	Lung/Chemoimmunotherapy	FMT from healthy/responders restores microbiota diversity, increases immune cell infiltration, and enhances chemoimmunotherapy efficacy (overcomes dysbiosis‐induced resistance).	FMT (donor‐dependent; restores beneficial taxa).	(X. Wang et al. (2025))
**Preclinical & animal models: Fecal microbiota transplantation (FMT) in animal models**	Chang et al. (2020) & follow‐ups – CRC‐bearing mice	CRC/FOLFOX (oxaliplatin + 5‐FU)	FMT prevents/alleviates chemotherapy‐induced intestinal mucositis and injury; improves recovery and implies better tolerance/efficacy (sensitivity role).	FMT (alleviates dysbiosis).	(Chang et al. (2020))
**Preclinical & animal models: Fecal microbiota transplantation (FMT) in animal models**	Multiple (e.g., reviewed 2022–2025) – GF/antibiotic‐pretreated tumor mice	CRC, various/Oxaliplatin, 5‐FU	FMT from responder donors reprograms microbiome to improve response; from resistant donors transfers resistance traits (causal dual role demonstrated).	FMT (strain‐specific engraftment).	(Xu et al. (2022); Gazzaniga and Kasper (2025); M. Xie et al. (2025))
**Human clinical trials: Observational studies–linking microbiome signatures to chemoresistance**	Systematic review of studies (Böhm et al. 2025) – Baseline fecal 16S/metagenomics	Ovarian, CRC, breast, gastric, lung, etc./Platinum, 5‐FU, taxanes, anthracyclines	Higher α‐diversity + specific taxa (e.g., *Angelakisella*, *Arenimonas*, *Roseburia*) linked to chemoresistance (ovarian); *F. nucleatum* enrichment in non‐responders (CRC, via autophagy); responders enriched with *Roseburia*, *Dorea*, *Bacteroides fragilis* (SCFA/immune support). Toxicity associations (e.g., diarrhea with *Klebsiella*).	Depletion of butyrate‐producers or enrichment of pathobionts (*F. nucleatum*, *Bacteroides* subsets).	(Böhm et al. (2025))
**Human clinical trials: Observational studies–linking microbiome signatures to chemoresistance**	Terrisse et al. (2021) & others – Breast/gynecological cohorts	Breast/Anthracyclines/taxanes	Specific signatures (e.g., *Coprococcus*, *Clostridiales*, *Bifidobacteriaceae*) predict better response; Firmicutes shifts linked to hematologic toxicity.	Taxa like *Eubacterium*, *Lachnospiraceae* (depletion = worse outcomes).	(Terrisse et al. (2021))
**Human clinical trials: Interventional–fecal microbiota transplantation**	Limited direct chemoresistance trials (mostly ICI‐refractory); exploratory extensions (e.g., NSCLC, CRC models)	Various/Chemo or chemo‐ICI	FMT is safe and restores diversity; improves chemoimmunotherapy outcomes in small cohorts by reversing dysbiosis; primarily proven for ICI but extends to chemo toxicity/response (e.g., reduced mucositis, better PFS). Causal proof is stronger in animals.	FMT (from healthy or responder donors).	(Kelly et al. (2023); Chong et al. (2024); Y. Wang et al. (2025))
**Human clinical trials: Interventional–probiotics, prebiotics, and synbiotics**	Meta‐analysis (2025) + RCTs (e.g., *L. rhamnosus*, multi‐strain) – CRC, breast, head/neck	CRC, breast/5‐FU, FOLFOX, taxanes	Probiotics/synbiotics reduce GI toxicities (e.g., diarrhea RR ↓, mucositis) and modestly improve QoL/response rates; prebiotics (e.g., XOS) enhance drug bioavailability and mitigate dysbiosis (sensitivity‐enhancing via SCFA boost).	*Lactobacillus* spp. (e.g., *rhamnosus*, *plantarum*), *Bifidobacterium*; xylooligosaccharides (XOS), inulin.	(Chen et al. (2023); Eslami et al. (2025))
**Human clinical trials: Interventional – dietary interventions**	RCTs (e.g., fiber/prebiotic‐enriched diets, resistant starch) – CRC, various	CRC/Capecitabine, oxaliplatin	High‐fiber/prebiotic diets increase butyrate/SCFAs, restore beneficial taxa, reduce toxicity, and improve response (e.g., prebiotic nanoparticles + capecitabine ↑ tumor inhibition 5%→72% in models, translated clinically).	Dietary fiber, resistant starch, inulin; prebiotic‐supplemented diets.	(Lam et al. (2021); Spencer et al. 2021)

Abbreviation: BAX, Bcl‐2‐associated protein X; CDKN1B, cyclin dependent kinase inhibitor 1B; FOLFOX, folinic acid, 5‐fluorouracil and oxaliplatin.

## 9 Discussion

There is strong evidence showing that the gut microbiota acts as a key controller of chemoresistance. The conventional understanding of chemotherapy as being a simple process involving the interaction between a drug and a cancerous cell is becoming increasingly outdated. Rather, it is essential to consider the tripartite relationship among the drug, the cancer, and the host's symbiotic microorganisms. There are several underlying mechanisms, including drug metabolism, immune modulation, metabolic product formation, and homeostasis maintenance. These have been well understood through the synthesis of preclinical and clinical research findings, paving the way for new avenues of treatment.

### 9.1 Synthesizing the Evidence: From Mechanism to Clinical Application

The leap from understanding the role of bacterial CDDs in contributing to gemcitabine resistance in vitro to examining the feasibility of microbiome‐based therapies for treating cancer patients showcases the field's rapid evolution. The biological rationale for the mechanisms by which a bacterium like *F. nucleatum* creates a microenvironment in which immunogenic stimuli are suppressed, or by which butyric acid alters gene expression via epigenetic mechanisms, is clearly evident. The importance of developing animal models to establish causation and assess the potential for transferring treatment response via FMT cannot be overemphasized (Spencer et al. 2021). The success of FMT in reversing resistance to immunotherapy in clinical trials provides strong encouragement for this line of research and the potential to use it as part of chemotherapy treatment strategies. FMT is a broad‐spectrum intervention, but concurrent development of more refined approaches, such as probiotics or dietary changes, may make these strategies more applicable and safer. A high‐fiber diet is associated with significant improvements in patient survival during immunotherapy (Spencer et al. 2021; Lam et al. 2021).

### 9.2 Challenges, Unanswered Questions, and Future Directions

While there is significant interest, the research area faces several hurdles that must be overcome to unlock the therapeutic value of microbiome‐based approaches in medicine. One of the main hurdles is the shift from correlation to causation in the case of humans. While many studies have found certain microorganisms correlated with particular outcomes, it may still be challenging to determine whether these microorganisms drive resistance or merely serve as indicators of the true cause, which may be a host condition (e.g., inflammation or a poor diet). Another significant hurdle is inter‐individual variability. Microbiomes are highly personalized, implying that a generalized probiotic or diet strategy will not work equally well for everyone.

Standardization represents an important issue in this field. For FMT, there is currently no consensus on donor screening, stool processing, or delivery method, all of which can affect treatment effectiveness. When it comes to probiotics, the available products vary widely in quality, and their bacterial strains, doses, and viability have not been clearly defined. Without standardization, research results will be difficult to compare, let alone formulate practice guidelines based on evidence. Safety is another key factor, especially when live bacteria are delivered via FMT or probiotics to patients with severe neutropenia or other forms of chemotherapy‐induced immunosuppression. While major safety concerns have thus far been infrequent, careful surveillance is still required.

This area will evolve toward personalization and precision. As new developments in omics (metagenomics, metatranscriptomics, and metabolomics) emerge, scientists will be able to achieve a deeper understanding of the role of bacteria. This means that we will no longer focus on detecting the types of bacteria present in patients' bodies, but rather on determining the roles these bacteria play in mediating chemoresistance. In other words, we will learn how to predict which patients need microbiome‐based interventions.

The generation of advanced probiotics (“bugs as drugs”) can serve as another fruitful direction of study. In this case, certain beneficial strains of bacteria with clearly understood mechanisms of action, such as powerful immunomodulatory effects or high SCFA production, would need to be isolated and developed into a pharmaceutical‐grade product. Additionally, it might be possible to modify bacteria to perform a specific task in the human body, such as producing an antitumor agent within the tumor microenvironment. As another approach, postbiotics might be considered: sterile byproducts of bacterial metabolism (e.g., SCFAs), which may have all the benefits of probiotics without any of the associated risks.

The overall goal is to bring microbiome therapy into routine oncology practice. In particular, one might consider screening patients' microbiomes at the time of diagnosis to predict their response to a specific type of chemotherapy. In cases where the microbiome is dysbiotic or a “nonresponder,” a targeted intervention with a specialized synbiotic product, a diet regimen, or FMT could be considered before starting chemotherapy. Thus, the whole concept could imply a shift in the perception of treatment from an exclusive focus on cancer to consideration of the human as a complex superorganism.

## 10 Conclusion and Future Perspectives

The interplay between the gut microbiota and cancer, and its role in chemoresistance, has marked a paradigm shift in cancer biology and treatment. It has been shown that the millions of microorganisms in the human body actively participate in disease development, and their activity can lead to therapeutic success or failure. In this regard, the current review provides a synthesis of the evidence that reveals both the adverse impact of some species, like *F. nucleatum*, which promote drug inactivation and cell survival, and the positive influence of other bacteria, including *Bifidobacterium* and *A. muciniphila*, on cancer treatment through enhanced immune response and production of favorable metabolites.

The mechanisms underlying these actions are highly diverse and include direct interactions between the gut microbiota and chemotherapeutic agents, extensive regulation of the host immune response, and intensive metabolic activities that affect the tumor microenvironment. Chemotherapy is likely to disrupt this complex system, leading to dysbiosis and increased gut permeability. However, this intricate mechanism also creates numerous opportunities to exploit the gut microbiota to enhance therapy efficiency. In this context, FMT, probiotic use, and targeted dietary modifications appear quite promising in preclinical studies and first‐in‐human trials.

The future appears promising for advancements in the field, though multiple issues still need to be addressed. First, it is crucial to gain a better mechanistic understanding of the roles of specific microbial taxa and functions in the effects of diverse cancer medications. To achieve this objective, a transition from correlative observations to functional multiomics approaches incorporating metagenomics, metatranscriptomics, and metabolomics should occur, alongside analysis of patient data. Second, further development of probiotics, as well as the design of rational microbial consortia based on this knowledge, will be required to overcome one‐size‐fits‐all treatment. Third, an interaction among nutrition, the microbiota, and cancer therapy remains in need of thorough investigation in clinical studies. Fourth, the creation of effective markers to help identify patients resistant to chemotherapy is highly important.

In conclusion, the GM is now a key component of overcoming chemoresistant cancer. An improved strategy that incorporates the complexity of the host–microbe–drug relationship will yield newer therapies that not only target the cancerous tumor but also help create a healthier microbial environment. Using these new findings in the clinic will take time; however, it will drastically change how we treat cancer patients by increasing the effectiveness and decreasing the toxicity of chemotherapy, thereby saving many lives.

## Author Contributions

**Hamed Tahmasebi:** conceptualization, software, investigation, writing – review and editing, writing – original draft. **Aisa Bahar:** investigation, writing – original draft, writing – review and editing. **Meisam Khazaei:** software, investigation, writing – original draft, writing – review and editing. **Mohammad Reza Arabestani:** data curation.

## Funding

The authors have nothing to report.

## Ethics Statement

The authors have nothing to report.

## Consent

The authors have nothing to report.

## Conflicts of Interest

None declared.

## Supporting information

Supporting File

## Data Availability

The data that support the findings of this study are available on request from the corresponding author. The data are not publicly available due to privacy or ethical restrictions.
