# Supplementary figures and images for: The Dual Role of the Gut Microbiota in Cancer Chemoresistance
Source: Microbiologyopen. 2026 Jul 8;15(4):e70357. doi: 10.1002/mbo3.70357 (PMC13345602; doi:10.1002/mbo3.70357)

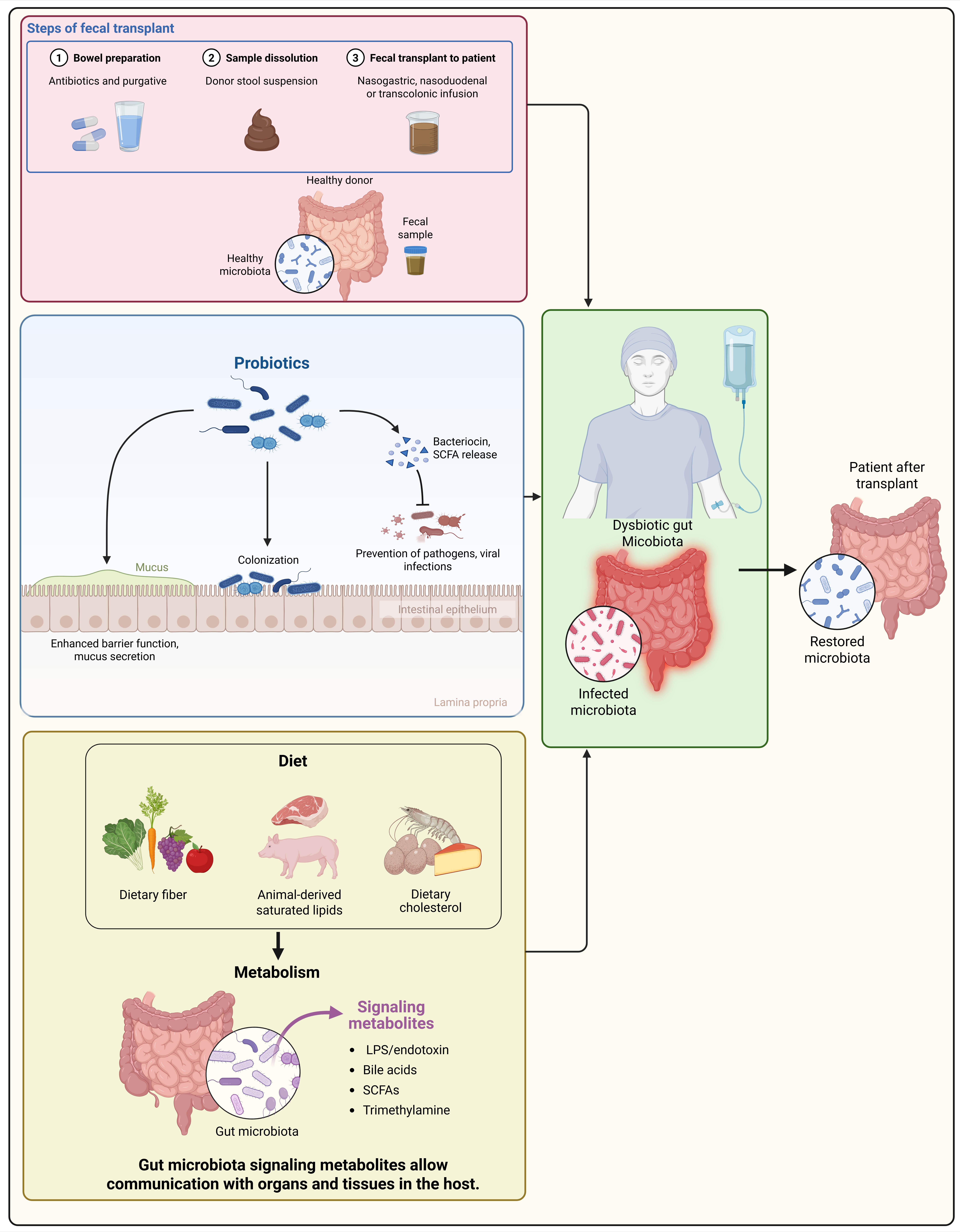

Supplement: Supplementary file 1 — Supporting File [file MBO3-15-e70357-s001.jpg]
